# Do catadromous thinlip grey mullet benefit from shifting to freshwater? A perspective from fatty acid signature analysis

**DOI:** 10.1007/s10695-024-01322-9

**Published:** 2024-02-27

**Authors:** Esmeralda Pereira, André Jorge, Bernardo Quintella, Marco Gomes da Silva, Pedro R. Almeida, Maria João Lança

**Affiliations:** 1 MARE–Centro de Ciências do Mar e do Ambiente/ARNET-Rede de Investigação Aquática, Universidade de Évora, Largo Dos Colegiais N.2, 7004-516 Évora, Portugal; 2 MED–Mediterranean Institute for Agriculture, Environment and Development/CHANGE–Global Change and Sustainability Institute, Universidade de Évora, Pólo da Mitra, Ap. 94, 7006-554 Évora, Portugal; 3 LAQV-REQUIMTE-Departamento de Química, Faculdade de Ciências E Tecnologia, Universidade Nova de Lisboa, Campus de Caparica, 2829-516 Caparica, Portugal; 4 MARE–Centro de Ciências do Mar e do Ambiente/ARNET-Rede de Investigação Aquática, Faculdade de Ciências da Universidade de Lisboa, Campo Grande, 1749-016 Lisbon, Portugal; 5 Departamento de Biologia Animal, Faculdade de Ciências da Universidade de Lisboa, Campo Grande, 1749-016 Lisbon, Portugal; 6 Departamento de Biologia, Escola de Ciências E Tecnologia, Universidade de Évora, Largo Dos Colegiais N.2, 7004-516 Évora, Portugal; 7 Departamento de Zootecnia, Escola de Ciências E Tecnologia, Universidade de Évora, Ap.94, 7006-554 Évora, Portugal

**Keywords:** Catadromy, Trophic migration, Mugilidae, FAME, GC/MS chromatography - Reproductive strategy

## Abstract

To investigate the potential benefits of the catadromous thinlip grey mullet (*Chelon ramada* Risso, 1827) migration to freshwater, the total lipid content and fatty acid (FA) profile of female’s muscle and gonads caught in both the estuary and river were analyzed. The freshwater contingent presented a higher body condition, greater muscle gross energy, and larger gonads with higher lipid reserves. These animals showed a muscle profile rich in C16:1n-7 and lower LC-PUFA that contrast with the higher relative amount of C18:1n-9, n-3 FA, and unsaturated LC-PUFA, such as C18 and C20 FA found in the estuarine contingent. The gonads of both contingents showed a constant and high relative amount of polyunsaturated FAs (PUFA, 37%). However, in terms of essential fatty acids (EFAs), the estuarine contingent had a higher relative amount of C18:2n-6, C20:4n-6, C20:5n-3, and C22:6n-3. On the other hand, the freshwater contingent showed a higher relative amount of n-3 FA precursors, namely C18:3n-3, and a still low relative amount of C22:6n-3. This suggests a mismatch between the metabolic omega-3 pathway and the physiological maturity stages, similar to a phenomenon of dormancy. In this sense, not all these individuals may reproduce annually, and the later stages of gonad development will require supplementary energy derived from feeding at the estuary. Thus, freshwater migration may promote a reproductive strategy enabling adults to take advantage of the warm and food-rich summer/autumn period, adjust spawning and juvenile appearance, and reduce the population’s exposure to habitat changes and/or stochastic events.

**Supplementary Information:**

The online version contains supplementary material available at 10.1007/s10695-024-01322-9.

## Introduction

Species movement between distinct environments requires major physiological and behavioral adaptations (Hinch et al. 2006). Despite migration’s high energetics and potential fitness costs, diadromous species developed a wide range of effective life-history strategies associated with different migratory behaviors (McDowall 1997). These behaviors are shaped and influenced by a complex interplay of internal and external factors. Internal factors can comprise genetic and ontogenic triggers, metabolic balance, and homing, while external factors include food availability, predator avoidance, pathogen/parasitic reduction, and seasonal changes (Lucas and Baras 2001; Wille and Klaassen 2023). From an evolutionary perspective, the natural selection process favors the maintenance of traits and response to stimuli that trigger behaviors leading to enhanced lifetime reproductive success (Lucas and Baras 2001; Brönmark et al. 2014).

Migration to different environments involves the exploitation of habitats with different productivity and energy levels, which can lead to differences in food sources, resulting in different levels of dietary lipid content (Koussoroplis et al. 2010; Carassou et al. 2017). The producers of marine food chains are unicellular algae rich in polyunsaturated fatty acid (PUFA), mainly from the n-3 family plus the marine microbiome (heterotrophic protists and bacteria), being the last one, the richest sources of fatty acids (FAs), particularly of essential n-3 PUFA. In opposition, freshwater food chain producers have a higher PUFA content from the n-6 family (Dalsgaard et al. 2003; Monroig and Kabeya 2018). These lipids and their constituent FA, especially the polyunsaturated of the n-3 and n-6 pathways, have important functions in physiological processes. They serve as an energy source, contribute to the structure of biological membranes, regulate the membrane function and fluidity, and act as precursors for important molecules such as thromboxane, prostaglandins, and leukotrienes (Tocher 2003; Alfaro et al. 2006; Lança et al. 2015). FAs are also involved in many other biological functions, including immune response, renal and cardiovascular functions, and reproduction, as they act as precursors for eicosanoids (Tocher 2010). For instance, essential fatty acids (EFAs) such as eicosapentaenoic acid (EPA, C20:5n-3), docosahexaenoic acid (DHA, C22:6n-3), and arachidonic acid (ARA, 20:4n-6) are known to play a crucial role in growth and survival at early life stages of both invertebrates (Goedkoop et al. 2007) and vertebrates (Bae et al. 2010; Xu et al. 2010; Paulsen et al. 2014). In addition, these FAs are important in fish reproduction, affecting factors such as fecundity, fertilization, egg viability, hatching rates, and larvae survival (Almansa et al. 2001; Mañanós et al. 2008; Dhurmeea et al. 2018).

The differences in productivity and energy between the systems can have significant effects on fish metabolism and reproductive success. Nevertheless, fish performance and fitness will ultimately depend on the ability of the specimen to obtain optimal dietary inclusion levels, a balance between FA families (n-3 and n-6), and the most appropriate FA chain- lengths (18-C, 20-C, or 22-C), which are species-specific (Kjesbu et al. 1991; Almansa et al. 2001; Sorbera et al. 2001; Mañanós et al. 2008; Xu et al. 2017) and vary accordingly with ontogeny (Tocher 2010). For instance, during oocyte maturation, changes in the FA composition and proportions of lipid classes in various tissues are strongly linked to their respective functions (McBride et al. 2015; Khajeh et al. 2017). Thus, dietary switches that result in distinct FA profiles can have a significant impact on the fish condition, biological quality of spawners, and reproductive potential (Sorbera et al. 2001; Gonzalez-Silvera et al. 2016; Tong et al. 2017; Capoccioni et al. 2018).

However, the role of different migratory behaviors and breeding strategies in fish metabolism and the lipid reserves’ quantity and quality remain poorly studied (Capoccioni et al. 2018; Ramos-Júdez et al. 2023), particularly among species with lower economic value, such as the catadromous thinlip grey mullet (*Chelon ramada* Risso, 1827). A highly abundant species distributed along the Northeastern Atlantic, the Mediterranean, and the Black Sea (Turan 2016) that spawn in the sea and undergo a somatic growth period that can occur in the estuary or often, but not always, in freshwater environment (Almeida 1996; McDowall 1997; Pereira et al. 2021).

In the Atlantic region, a fraction of the *C. ramada* population is known to perform an extensive trophic migration to freshwater, which usually starts in March and can extend until November (Pereira et al. 2021). During this period of somatic growth, this euryhaline species shows a high osmoregulatory capacity and feeding plasticity (specialized feeding apparatus) that provide them a unique ability to exploit spatially variable resources (Lebreton et al. 2011; Cardona 2016; Carassou et al. 2017). Since *C. ramada* frequently moves through different habitats during its life cycle, a better understanding of the implications of individual behavior and habitat use on specimen condition and metabolism is crucial to identify the proximate and ultimate causes underlying this life-history strategy. Therefore, this study aims to investigate the benefits of *C. ramada* trophic migration to freshwater by analyzing and comparing the muscle and gonads’ composition and FA profiles associated with estuarine residency versus freshwater migration. We hypothesized that distinct behavioral profiles (i.e., contingents) associated with migrations between environments (estuarine vs freshwater) lead to differences in fish condition, energy allocation, and FA profiles of muscle and gonads, which are possibly linked to distinct breeding strategies.

## Material and methods

### Fish samples

**Fig. 1:**
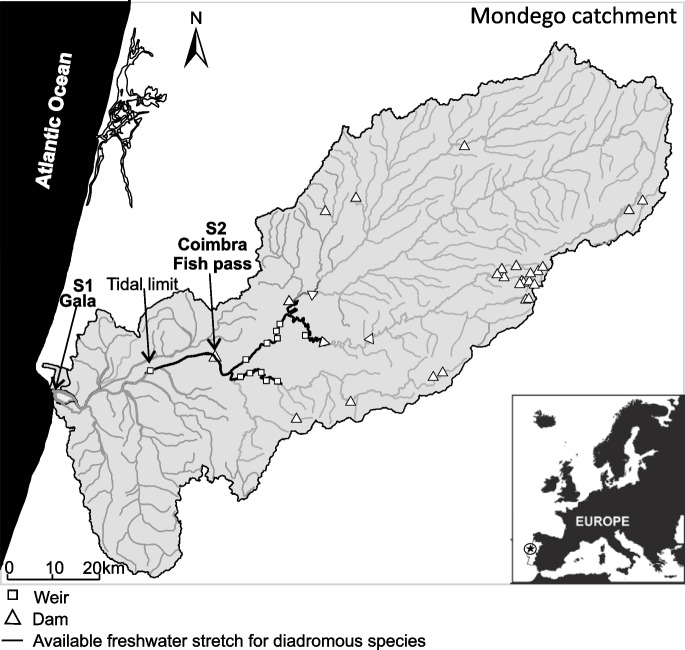
River Mondego Basin (central Portugal) with the location of the sampling sites in the estuary (S1) and freshwater (S2) environments

During the seasonal trophic migration of 2019, a total of three sampling campaigns were carried out in the Mondego river basin (central Portugal), covering species’ early period of trophic migration (spring) and pre-spawning migration (late summer). Spring sampling (30th of April) was carried out exclusively in the Mondego estuary (S1: 40°08′28.9″N 8°51′15.7″W), using a trammel net operated by commercial fishermen, and it established the specimen’s condition preceding the feeding and growing period in continental waters. In summer and to cover both species’ migratory profiles (i.e., freshwater trophic migration vs estuarine residency), sampling was carried out either on the Mondego estuary (S1: 20th of August) and in an upstream river stretch (S2: 40°12′53.7″N 8°26′25.6″W, 24th of September) (Fig. 1). In freshwater, the animals were trapped inside a fish pass (Coimbra Dam, Pereira et al. 2021). Summer sampling was performed with a time lag between the estuarine and freshwater environments to reduce the potential pre-spawning mixture of both profiles in the estuary. From each sampling campaign, 50 animals were transported alive in an oxygen-supplied tank (500L) to the laboratory, where they were maintained in adequate life support systems (LSS) (i.e., water quality, oxygenation until further euthanasia and processing). Captured specimens were euthanized individually by contusion, the gonad was macroscopically examined, and females were immediately processed until 20 adult female *C. ramada* specimens were sampled. Females were chosen since a higher energetic investment in gonad development and maturation is required. Among the storage tissues, the muscle was selected as it reflects changes in the FA profile following a shift in the diet in a short period of time, as 30 days (Gonzalez-Silvera et al. 2016). Considering that the lipids stored throughout the trophic season can be mobilized for reproduction, the gonads were also analyzed.

For each female, data on total body length (*TL*, nearest millimeter), total body mass (*TW*, nearest g), eviscerated body mass (*EW*, nearest g), liver total mass (*LW*, nearest g), gonad total mass (G*W*, nearest g), and ovary developmental stages (according to Bruslé, 1982) were recorded. The skin was removed, and the muscle tissue was exposed. The entire white muscle in the proximity of the mid-dorsal line, in the left flank of the animal close to the dorsal fin, was collected, washed with physiologic saline, fractionated in three replicates of 5 g, well-homogenized to be representative of the whole area and immediately stored at − 80 °C until laboratorial processing. The gonads were collected and similarly washed, fractionated in three replicates of 5 g, and stored at − 80 °C until laboratorial processing.

The remaining animals that were alive were transported to the sampling location and released. All animal handling procedures were carried out in strict accordance with the recommendations present in the Guide for the Care and Use of Laboratory Animals of European Union 62/2010, in Portugal under DL no. 192/92, Portaria no. 1005/92, and DL 113/2013 and approved by the University of Évora ethics committee (ORBEA). Euthanasia was performed following the recommendations of the official regulations previously mentioned.

### Proximate composition analysis

The moisture content of each tissue sample was determined according to IPQ (1991) by drying at 105°C until weight stabilization. Dry samples were homogenized and kept sealed at room temperature. Muscle crude protein content was determined by combustion (LECO, FP-528) according to AOAC (1990) and considering a Kjeldahl nitrogen of 6.25. Muscle gross energy content was measured using an automatic oxygen bomb calorimeter (Parr 6400, U.S.A.) according to ISO 9831. Results were converted from megajoules (MJ) per kg of dry matter to kilocalories (kcal) per 100 g of wet weight.

### Determination of fatty acid profiles

Fatty acid methyl esters (FAMEs) of total lipids present in muscle and gonad were extracted through Dionex 100 accelerated solvent extractor (ASE), using a Folch’s method solvent mixture (Folch et al. 1957). Aliquots of homogenized and lyophilized samples with 0.1 g and 0.05 g (± 0,005 g), respectively, were pulverized in an aluminum mortar with a stainless-steel pestle, both cooled in liquid nitrogen. The tissue powder was combined with a matrix drying agent (Diatomaceous Earth, hydro matrix Varian, P/N 049458) to a total of 2.0 g and transferred to a 10-mL stainless steel extraction cell. The total lipids were then extracted with a mixture of chloroform/methanol (60:40 V:V) (Merck; Darmstadt, Germany) at 100 °C and at 13.8 MPa. Both extraction solvents were residue-analysis grade and were treated with 100 mg/L BHT (3,5-Di-*tert*-butyl-4-hydroxytoluene, Merck, Darmstadt, Germany) as an antioxidant. Two static extraction cycles were carried out during a 5-min period each. The crude extract was then concentrated under a stream of nitrogen using a Rotavapor R-114 and water bath B-480 apparatus (Buchi, Switzerland) set at a bath temperature of 40 °C and weighed. The crude extract was reconstituted in 1 mL of NaOH 0.5 N at 70 °C for 15 min. FAMEs were then prepared with a fresh open boron-trifluoride-methanol (14 g BF_3_ L^−1^ CH_3_OH, Merk-Schuchardt, Germany) bottle, using sealed Teflon-lined screw-top tubes to produce FAME according to the procedure of Morrison and Smith (1964) and 1 µL of the recovered organic phase was analyzed by a GC/MS system. Further details are described in Jorge et al. (2021).

FAMEs were analyzed on a GC/MS system consisting of a Bruker GC 456 with a Bruker mass selective detector Scion TQ. The system was equipped with an automatic sampler injector and a CTC analysis autosampler CombiPal. Data were acquired with MSWS 8.2 Bruker and analyzed with Bruker MS Data Review 8.0. Chromatographic separation was achieved on a ZB-WAX Plus 60 m × 0.32 mm i.d., 1.0-µm film thickness (d_f_) capillary column supplied by Phenomenex, Torrance, CA, USA. The chromatographic conditions were helium as carrier gas at a constant flow of 2.0 mL/min; injector operated in splitless mode for 1 min, at 270 °C; MS interface 240 °C; and MS source 220 °C. The oven temperature was held at 120 °C for 5 min and then increased from 120 to 250 °C with a ramping rate of 5 °C/min to 250 °C where it was maintained for 59 min. The peaks and their respective MS were analyzed by electronic impact at 70 eV, within the range of m/z = 40 to 450 Da. The FAME standards used were 37-component FAME MIX and Bacterial Acid Methyl Ester (BAME) MIX 26 (Supelco), with 26 components. The integrated chromatogram values for each FAME (and hence the correspondent FA) were expressed as a percentage of the total sum of FA identified to eliminate concentration effects. FAs were designated according to the International Union of Pure and Applied Chemistry (IUPAC) nomenclature for carbon chain length: number of double bonds and position of the double bond closest to the omega carbon. FAs were expressed as a percentage of the total FA identified and were normalized using an arcsine transformation (Fowler et al. 2002).

### Data analysis

The gonadosomatic index (GSI, I_g_ = 100 × gonad weight (g)/eviscerated weight (g)) and the hepatosomatic index (HSI, I_H_ = 100 × (liver weight(g)/eviscerated weight(g)) were calculated. Additionally, the relative condition factor (*K*′), an extension of Fulton’s condition factor developed by Ricker (1975), was estimated following the formula:

$${K'}=\frac{EW{}}{TL^{b}}\times 100,$$

where EW and TL are the observed eviscerated body weight (in g) and total length (in cm) of a mullet. Based on the slope of mullet eviscerated weight–length relationship for Mondego basin (unpublished data), *b* was considered 2.78.

Differences in biometric and proximate composition between seasons and across sites were analyzed through the non-parametric Kruskal–Wallis test with Bonferroni-adjusted Dunn’s pairwise comparisons. For each tissue, significant differences in the main FA groups along the migratory season (factor “Season,” two fixed levels: “spring,” “summer”) and sampling site (nested factor: “Site,” two fixed levels: "estuary,” “river”) were inferred based on the FA classes and families (∑SFA, ∑MUFA, ∑PUFA, ∑HUFA, ∑odd FA, ∑ISO FA, ∑Ante FA, ∑n-3, ∑n-6, ∑n-1–4-5, ∑n-7, ∑n-9) and tested through the permutational multivariate analysis of variance (PERMANOVA). Additionally, for the muscle tissue, differences based on the trophic markers, more precisely microbial, macroalgae, diatoms, and dinoflagellates (see Supplementary Material 1), were analyzed. Principal coordinate analysis (PCO) based on Euclidean distance matrix and similarity of percentages analysis (SIMPER) were performed to investigate the variation in fatty acid signatures and to identify the classes, families, and trophic markers that strongly influenced this variation. PERMANOVA, PCO, and SIMPER were carried out using the software PRIMER v6.0 (Anderson et al. 2008), and the remaining statistical analyses were performed in Rstudio (v 2023.03.0R). Differences were considered significant at *P* < 0.05.

## Results

### Biometric parameters and proximate composition

Females sampled in spring had a mean total length of 362.0 ± 28.35 mm, a mean eviscerated weight of 314.1 ± 79.71 g, and Fulton’s condition factor, HSI, and GSI, with a mean value of 1.38 ± 0.07, 0.90 ± 0.21, and 0.65 ± 0.24, respectively. In summer, significant differences in the biometric parameters were observed between sampling locations (Table 1). While the females from the estuary had a similar mean length and a relative increase in body weight (380.28 ± 92.08 g) when compared with the spring sampling, the animals from the freshwater contingent were significantly smaller in size (χ^2^ = 15.20;* P* < 0.001) and weight (χ^2^_(TW)_ = 9.05; *P* < 0.05; χ^2^_(EW)_ = 7.90; *P* < 0.05). Still, they displayed the highest mean value of GSI (1.70 ± 1.16; χ^2^ = 16.09; *P* < 0.001) and Fulton’s condition (1.64 ± 0.29; χ^2^ = 28.25; *P* < 0.001) (Table 1).

**Table 1:** Biometric data of *Chelon ramada* females sampled in the Mondego River basin according to the season and site of capture. Mean ± standard deviation is presented. Values with different superscripts within rows are statistically different, *P* < 0.05

	Spring	Summer	
	Estuary (*n* = 20)	Estuary (*n* = 20)	River (*n* = 20)
Total body length (mm)	362.05 ± 28.35^a^	362.80 ± 28.81^a^	321.75 ± 33.44^b^
Total body mass (g)	345.99 ± 89.01^ab^	380.28 ± 92.08^a^	284.58 ± 79.46^b^
Eviscerated body mass (g)	314.15 ± 79.71^ab^	341.18 ± 76.74^a^	266.81 ± 78.90^b^
Gonadosomatic index (GSI)	0.65 ± 0.24^a^	0.83 ± 0.13^ab^	1.70 ± 1.16^b^
Hepatosomatic index (HSI)	0.90 ± 0.21^a^	1.06 ± 0.13^a^	0.99 ± 0.40^a^
Fulton Index	1.38 ± 0.07^a^	1.50 ± 0.09^b^	1.64 ± 0.29^c^
Gonad maturation stage (%)			
II	0.30	0.05	0.05
III	0.70	0.95	0.30
IV	–	–	0.65

**Fig. 2:**
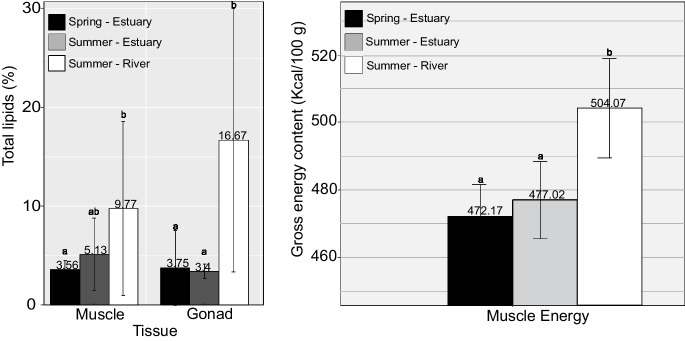
Variation in tissue total lipid content (%, dry weight) and muscle’s gross energy content (kcal/100 g dry weight) of *Chelon ramada* females in the beginning (early spring) and end (late summer) of the trophic migration season in two locations (estuary and river). Data is presented as mean ± standard deviation, for *N* = 20 for each sampling campaign. Different superscript letters identified significant differences within rows (KW), *P* < 0.05

The muscle of *C. ramada* in the spring was characterized by an average water content of approximately 77.94 ± 1.26% (and consequently 22 ± 1.26% of dry matter), almost 18.84 ± 0.86% of crude protein (dry weight), 3.56 ± 0.96% total lipids (dry weight), and a gross energy content of 472.17 ± 9.02 kcal/100 g (Table 2, Fig. 2). No significant differences were found in muscle water content (χ^2^ = 1.67; *P* = 0.43) throughout the season (spring vs. summer) and between sites (estuary vs. freshwater). During summer, no significant differences were found in muscle total lipids (*P* = 0.056) between the estuarine and freshwater contingents. The females from the estuary displayed a significant increase in dry matter (24.23 ± 1.43% dry weight, χ^2^ = 18.24; *P* < 0.001) and total crude protein (20.53 ± 0.78% dry weight, χ^2^ = 27.20; *P* < 0.001), while the ones captured in freshwater had a significant increase in gross energy content (504.07 ± 14.71 kcal/100 g, χ^2^ = 34.52; *P* < 0.001).

**Table 2:** Proximate composition (mean ± standard deviation, % of dry weight) of muscle and gonads of *Chelon ramada* females collected at the beginning (early spring) and end (late summer) of the trophic migratory season in the estuary and river (freshwater section). Different letters in superscripts identified significant differences within rows (KW), *P* < 0.05

		Spring	Summer	
		Estuary	Estuary	River
Muscle	Water content (%)	77.94 ± 1.26^a^	75.77 ± 1.43^a^	77.46 ± 1.98^a^
	Dry matter (%)	22.06 ± 1.26^a^	24.23 ± 1.43^b^	22.54 ± 1.98^a^
	Total crude protein (%)	18.84 ± 0.86^a^	20.53 ± 0.78^b^	18.50 ± 1.66^c^
Gonad	Water content (%)	81.65 ± 0.88^a^	78.88 ± 0.82^b^	74.22 ± 6.08^c^
	Dry matter (%)	18.35 ± 0.88^a^	21.12 ± 0.82^b^	25.78 ± 6.08^c^

The gonad tissue showed significant differences in terms of water content (χ^2^ = 34.47; *P* < 0.001), dry matter (χ^2^ = 18.24; *P* < 0.001), and lipids (χ^2^ = 31.47; *P* < 0.001) between spring and summer. A decrease in water content was observed from the beginning of the trophic migration in early spring (81.65 ± 0.88%) to the end (late summer), with the lowest values being recorded in freshwater (74.22 ± 6.08% dry weight). This contingent displayed the highest content of dry matter (25.78 ± 6.08% dry weight) and a significantly higher content of total lipids (16.67 ± 13.33% dry weight) (Table 2, Fig. 2).

### Lipid content and fatty acid profile

#### Muscle

**Fig. 3:**
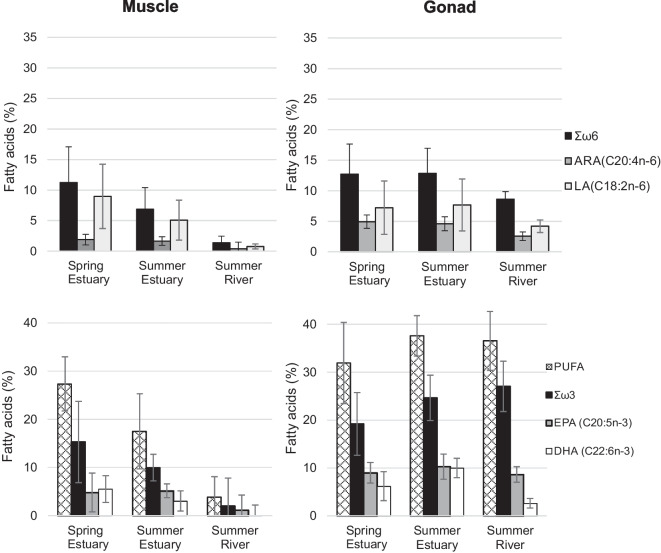
Total fatty acid content (%) of ∑n-6, ARA, LA (C18:2 n-6), ∑n-3, EPA, and DHA in the muscle and gonads of the *Chelon ramada* females sampled in the beginning (early spring) and end (late summer) of the trophic migration season in the estuary and river habitats. Data is presented as mean ± standard deviation, for *N* = 20 for each sampling campaign

**Fig. 4:**
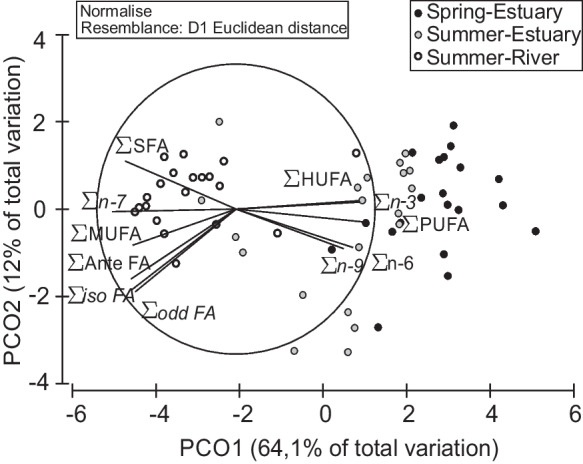
Principal coordinate analysis of fatty acid classes and families in the muscle of female *Chelon ramada* sampled at the beginning (early spring) and end (late summer) of the trophic migration season in estuary and river habitats

Muscle FA profile revealed significant differences between spring and summer samples (PERMANOVA, Pseudo-F = 22.6; *P* = 0.001, 999 permutations). In spring, the muscle presented a smaller content of saturated FAs (SFA, 44.63%) and the highest relative amount of polyunsaturated FAs (PUFA, 27.35%), with highly unsaturated FAs mostly represented (HUFA, 18.09%) (Table 3). In summer muscles, the most evident result was the reduced PUFA and HUFA in the freshwater contingent (3.89% and 2.89%, respectively), against the relative higher amount observed in the estuarine contingent (17.49% and 12.33%, respectively). Concerning FA families, a significant difference was observed between individuals sampled in the estuary and freshwater in late summer (Permanova: Pseudo-F = 19.4; *P* = 0.001, 999 permutations). Our results revealed that the estuarine contingent had a higher relative amount of the n-3 FA family (9.95%), when compared with the 2.05% observed in the freshwater contingent. Contributing to these results are mainly the relative amount of EPA, DHA, stearidonic acid (SDA, C18:4n-3), and docosapentaenoic acid (DPA, C22:5 n-3) (Table 3, Fig. 3). The PCO analysis conducted to complement PERMANOVA and SIMPER empathize the differences in the FA profile of both contingents (Fig. 4). The muscle FA profile of the freshwater contingent revealed a high relative amount of the n-7 FA family (29.31%), primarily associated with palmitoleic acid (C16:1n-7), which corresponded to 26.93% of total identified FAs. In contrast, the n-9 FA family had a higher relative amount in the muscle profile of the estuarine contingent (12.78%), which was attributed to the relative amount of oleic acid (C18:1n-9) MUFA (11.94%) (Table 3).

**Table 3:** Fatty acid composition (% of total fatty acids) of *Chelon ramada*’s muscle sampled in the beginning (early spring) and end (late summer) of the trophic migration season in the estuary and river habitats. Data is presented as mean ± standard deviation, for *N* = 20 for each sampling campaign. FA not detected identified as n.d

Muscle	Spring	Summer	
Fatty acids	Estuary	Estuary	River
C12:0	0.42 ± 0.18	0.42 ± 0.18	0.26 ± 0.30
C14:0	4.94 ± 2.65	9.60 ± 4.05	13.46 ± 2.35
C15:0	0.66 ± 0.12	0.87 ± 0.23	0.91 ± 0.19
C16:0	29.73 ± 4.45	36.13 ± 4.13	40.40 ± 2.33
C17:0	0.38 ± 0.12	0.43 ± 0.15	0.42 ± 0.07
C18:0	8.28 ± 2.05	7.73 ± 2.30	4.86 ± 1.21
C19:0	0.07 ± 0.06	0.11 ± 0.06	0.06 ± 0.06
C20:0	0.12 ± 0.09	0.08 ± 0.05	0.06 ± 0.04
**∑SFA**	**44.63 ± 6.06**	**55.45 ± 5.87**	**60.47 ± 3.26**
i-C14:0	0.02 ± 0.02	0.05 ± 0.03	0.10 ± 0.04
i-C15:0	0.25 ± 0.10	0.39 ± 0.20	0.38 ± 0.12
i-C16:0	0.09 ± 0.04	0.14 ± 0.05	0.15 ± 0.04
i-C17:0	0.16 ± 0.08	0.32 ± 0.15	0.33 ± 0.08
**∑iso**	**0.52 ± 0.20**	**0.90 ± 0.36**	**0.95 ± 0.24**
a-C15:0	0.06 ± 0.19	0.09 ± 0.11	0.13 ± 0.05
**∑ante-iso**	**0.06 ± 0.19**	**0.09 ± 0.11**	**0.13 ± 0.05**
C14:1 n-5	0.52 ± 0.26	0.19 ± 0.20	0.19 ± 0.23
C16:1 n-7	8.02 ± 3.98	11.02 ± 4.02	26.93 ± 4.62
C17:1 n-7	0.09 ± 0.06	0.12 ± 0.09	0.18 ± 0.07
C18:1 n-9	15.08 ± 3.99	11.94 ± 3.21	4.40 ± 1.78
C18:1 n-7	2.89 ± 0.75	1.96 ± 0.71	2.21 ± 0.60
20:1 n-9	0.54 ± 0.31	0.38 ± 0.32	0.13 ± 0.08
C20:1 n-9	0.31 ± 0.18	0.45 ± 0.57	0.52 ± 0.20
**ΣMUFA**	**27.44 ± 3.12**	**26.07 ± 4.72**	**34.55 ± 3.27**
C16:2 n-6	0.06 ± 0.06	n.d	0.22 ± 0.39
C16:3 n-4	0.41 ± 0.35	0.22 ± 0.18	0.41 ± 0.37
C16:4 n-1	0.41 ± 0.41	0.46 ± 0.43	0.04 ± 0.06
C18:2 n-6	8.97 ± 5.270	5.08 ± 3.27	0.78 ± 0.40
C18:3 n-3	1.40 ± 0.56	0.40 ± 0.32	0.54 ± 0.64
C18:4 n-3	2.48 ± 1.25	0.93 ± 0.57	0.16 ± 0.28
20:2 n-6	0.23 ± 0.24	0.08 ± 0.09	n.d
C20:4 n-6	1.89 ± 0.70	1.66 ± 1.06	0.40 ± 0.62
20:4 n-3	0.24 ± 0.19	0.10 ± 0.09	0.01 ± 0.05
C20:5 n-3	4.82 ± 1.42	5.15 ± 3.11	1.18 ± 1.60
C22:5 n-6	0.07 ± 0.14	0.04 ± 0.06	n.d
C22:5 n-3	0.87 ± 0.44	0.34 ± 0.32	0.06 ± 0.18
C22:6 n-3	5.50 ± 2.09	3.03 ± 2.13	0.09 ± 0.29
**ΣPUFA**	**27.35 ± 5.61**	**17.49 ± 7.80**	**3.89 ± 4.19**
**ΣHUFA**	**18.09 ± 3.05**	**12.33 ± 7.07**	**2.89 ± 3.74**
Σn-3	15.31 ± 2.77	9.95 ± 5.75	2.05 ± 2.90
Σn-6	11.22 ± 5.84	6.86 ± 3.54	1.39 ± 1.05
Σn-7	10.99 ± 4.42	13.10 ± 3.83	29.31 ± 4.83
Σn-9	15.93 ± 4.18	12.78 ± 3.44	5.05 ± 1.71
EPA/DHA	1.04 ± 0.61	3.50 ± 8.03	0.63 ± 1.97
ARA/DHA	0.43 ± 0.21	0.31 ± 0.10	0.32 ± 0.17

**Fig. 5:**
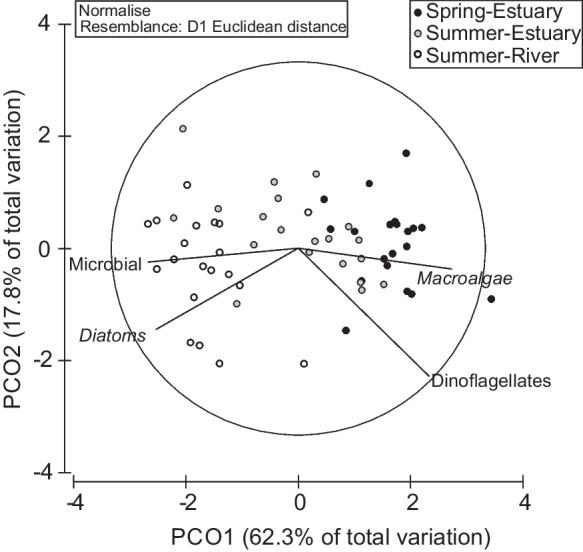
Principal coordinate analysis of fatty acid trophic markers in the muscle of female *Chelon ramada*

When considering the trophic markers, a clear segregation between the estuary and freshwater muscle FA profiles was observed (PERMANOVA: Pseudo-F = 12.86; *P* = 0.001; 999 permutations). In the estuarine contingent, regardless of the season, *C. ramada* muscle displayed a diet marked by macroalgae and phytoplankton, especially dinoflagellates. Conversely, in the freshwater contingent, *C. ramada* diet is characterized by microalgae, primarily diatoms, and FA of microbial origin (Fig. 5). Considering the FAs that integrate the group of macroalgae trophic markers, the ones that contributed the most to the dissimilarity between sites was the EPA (13.55%), which had a reduced relative mount in freshwater (1.18% compared to 4.82% and 5.15% in spring and summer estuary, respectively), followed by ∑C20 PUFA (13.14%), as well as the ratio ∑C16 PUFA/∑C18 PUFA (12.09%), that highlights the higher relative amount of C20 PUFA and C18 PUFA in the estuary. From the trophic marker set for dinoflagellates, EPA (18.38%), oleic acid (15.17%), and DHA (13.52%) contributed, among others, firmly to the dissimilarities found, and their relative amount were consistently lower in freshwater contingent. In terms of microalgae, the FA EPA (19.73%), C16:2n-6 (18.04%), and the ratio ∑C16/∑C18 (17.32%) had the higher contribution to the dissimilarity between river and estuarine contingents and reflected the lower EPA but higher C16 FA relative amount compared to longer-chain FA.

#### Gonad

Fatty acid composition of *C. ramada* gonads varied between the beginning and the end of the trophic migration season (Permanova, Pseudo-F = 5.99; *P* = 0.002, 999 permutations). Spring gonads exhibited the highest value of SFA (47.10%), as well as lower values of both PUFA and HUFA relative amounts (31.92 and 24.16%, respectively) when compared with those collected in late summer (Table 4).

**Table 4:** Fatty acid composition (% of total fatty acids) of female *Chelon ramada*’s gonads sampled in the beginning (early spring) and end (late summer) of the trophic migration season in estuary and river habitats. Data is presented as mean ± standard deviation, for *N* = 20 for each sampling campaign. FA not detected identified as n.d

Fatty acids	Spring	Summer	
	Estuary	Estuary	River
C12:0	0.17 ± 0.08	0.60 ± 0.18	0.18 ± 0.16
C14:0	1.54 ± 0.91	1.93 ± 1.27	3.31 ± 0.53
C15:0	0.61 ± 0.32	0.67 ± 0.68	0.32 ± 0.06
C16:0	30.42 ± 9.30	24.92 ± 2.89	21.95 ± 4.77
C17:0	0.54 ± 0.25	0.58 ± 0.15	0.35 ± 0.07
C18:0	13.55 ± 3.63	10.94 ± 2.44	5.08 ± 1.37
C19:0	0.18 ± 0.17	0.24 ± 0.10	0.03 ± 0.05
C20:0	0.10 ± 0.08	0.11 ± 0.07	0.04 ± 0.03
**∑SFA**	**47.10 ± 11.63**	**39.97 ± 5.26**	**31.25 ± 6.25**
i-C15:0	0.05 ± 0.05	0.13 ± 0.10	0.16 ± 0.08
i-C16:0	0.11 ± 0.08	0.16 ± 0.07	0.03 ± 0.03
i-17:0	0.30 ± 0.19	0.40 ± 0.19	0.21 ± 0.12
**∑i**	**0.46 ± 10.27**	**0.68 ± 10.32**	**0.40 ± 10.15**
C16:1 n-7	3.25 ± 1.49	3.72 ± 2.18	20.24 ± 5.09
C17:1 n-7	0.01 ± 0.03	0.01 ± 0.04	0.06 ± 0.04
C18:1 n-5	n.d	0.01 ± 0.03	0.12 ± 0.09
C18:1 n-9	13.73 ± 3.87	15.12 ± 8.20	5.65 ± 1.57
C18:1 n-7	2.75 ± 0.66	2.42 ± 0.50	5.16 ± 0.86
C20:1 n-9	0.60 ± 0.46	0.42 ± 0.17	0.14 ± 0.12
C20:1 n-9t	0.18 ± 0.17	0.07 ± 0.10	0.46 ± 0.20
**ΣMUFA**	**20.52 ± 4.29**	**21.77 ± 7.83**	**31.82 ± 4.95**
C16:2 n-6	0	0	0.26 ± 0.33
C16:3 n-4	0.03 ± 0.06	0.09 ± 0.20	0.87 ± 0.47
C16:3 n-3	0	0	0.65 ± 0.46
C18:2 n-6	7.22 ± 4.36	7.66 ± 4.26	4.19 ± 1.02
C18:2 n-3	0	0.01 ± 0.04	1.15 ± 0.44
C18:3 n-6	0	0	0.71 ± 0.32
C18:3 n-3	0.81 ± 0.64	0.56 ± 0.46	6.62 ± 3.37
C18:4 n-3	1.21 ± 1.35	0.85 ± 0.69	1.52 ± 0.57
C20:2 n-6	0.54 ± 0.54	0.47 ± 0.23	0.11 ± 0.07
C20:3 n-6	0	0.08 ± 0.09	0.43 ± 0.17
C20:4 n-6	4.94 ± 1.09	4.59 ± 1.17	2.55 ± 0.69
C20:3 n-3	0	0.01 ± 0.04	0.43 ± 0.37
C20:4 n-3	0.18 ± 0.19	0.22 ± 0.10	0.91 ± 0.52
C20:5 n-3	8.99 ± 2.15	1.28 ± 2.62	8.64 ± 1.65
C22:4 n-6	0	0.01 ± 0.02	0.25 ± 0.13
C22:5 n-6	0	0.05 ± 0.10	0.11 ± 0.13
C22:5 n-3	1.81 ± 1	2.72 ± 0.62	4.46 ± 1.20
C22:6 n-3	6.19 ± 3.02	9.99 ± 2.03	2.64 ± 1.03
**ΣPUFA**	**31.92 ± 8.46**	**37.58 ± 4.22**	**36.53 ± 6.15**
**ΣHUFA**	**24.16 ± 6.50**	**29.44 ± 5.56**	**30.82 ± 5.48**
Σn-3	19.19 ± 6.54	24.64 ± 4.74	27.03 ± 5.24
Σn-6	12.71 ± 4.93	12.85 ± 4.12	8.63 ± 1.23
Σn-7	6.01 ± 1.87	6.15 ± 2.55	25.46 ± 5.46
Σn-9	14.51 ± 4.32	15.62 ± 8.21	6.24 ± 1.59
EPA/DHA	5.87 ± 5.43	1.03 ± 0.21	3.63 ± 1.27
ARA/DHA	0.59 ± 0.21	0.46 ± 0.10	0.30 + 0.07

**Fig. 6:**
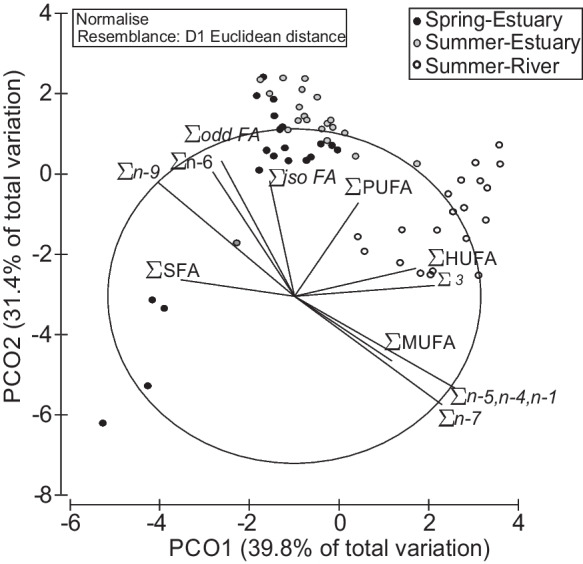
Principal coordinate analysis of fatty acid classes and families in the gonads of female *Chelon ramada* sampled at the beginning (early spring) and end (late summer) of the trophic migration season in estuary and river habitats

When considering the summer animals sampled in both environments (estuary and river), significant differences were observed between contingents (Permanova: Pseudo-F = 15.9; *P* = 0.001, 999 permutations). For the dissimilarities observed (25.09%), there was a high contribution both from FA families n-7 (16%) and n-9 (14%), but also from the MUFA class (12%) that were attributed to the high expressions of the palmitoleic acid and oleic acid, respectively (Fig. 6). The freshwater contingent showed a higher expression of MUFA (31.82%) compared to the estuary (21.77%) and it was also the same two FA that contributed to differences, namely with C16:1n-7 (20.24%) that accounts for 63% of the MUFA and for the 79% of n-7 FA identified. While in the estuary, the oleic acid (15.12%) represented 69% of the MUFA and 96% of n-9 FA observed (Table 4, Fig. 3).

Regarding unsaturated FA in the estuary or the freshwater contingent, PUFA and HUFA values are around 37% and 30%, respectively. The analysis of the composition of the PUFA class reveals that in the gonads of the spring animals, 59.55% of the PUFA corresponded to n-3 FA (Table 3). In summer animals, this value reaches 64.92% in the estuary and 65.37% in the freshwater contingent. Although in summer animals PUFA and HUFA relative amounts were similar, the FA profile analysis revealed that the EPA contributed to 52.88% of the estuary contingent and 58.12% in freshwater contingent for the total of n-3 gonad’s profile. As far as DHA is concerned, it appears that for the total n-3 FA gonad’s profile, it contributes 36.49% in the spring profile, 30.76% in the estuary profile in the summer, but only 4.43% in the freshwater profile. Hence, while relative amounts of EPA remained constant throughout the study (8.64 to 8.99%), the FA profile in freshwater showed a lower relative amount of DHA (2.64% vs 9.99%) and a higher ratio of EPA/DHA (3.63% vs 1.03%). Another important aspect related to the HUFA in the freshwater FA profile was the notable relative amount of α-linolenic acid (ALA, C18:3 n-3) (6.62%), as well as DPA (4.46%), and lower levels of arachidonic acid (ARA, C20:4 n-6) (2.55%) (Table 4; Fig. 3).

## Discussion

In the present work, it was hypothesized that different *C. ramada* migratory behaviors (estuarine residence *vs.* freshwater trophic migration), associated with distinct diet sources, will proximally lead to differences in fish condition, energy allocation, and FA profiles of muscle and gonads. These differences reflect species’ behavioral plasticity and are, ultimately, linked to specific metabolic conditions gathered in freshwater that contribute to species’ success. Our findings provide evidence that *C. ramada* specimens, which moved to freshwater, displayed a higher body condition associated with a higher muscle gross energy content and larger gonads, with a higher total lipid reserve that constitutes 16% of gonads’ dry weight. In addition, the decrease in water content, especially in the gonads, suggests a reproductive investment. The ovaries of fish are highly lipid-rich, with FA incorporated into the oocyte’s yolk to supply the embryo’s development (Adams 1999). This biological mechanism ultimately enhances the overall reproductive success of species through two advantageous situations: either by promoting the production of a greater number of oocytes, ensuring higher fecundity, or by facilitating the production of larger oocytes, which significantly improve larval survival rates and recruitment (Mayer et al. 1988;Kjesbu et al., 1991; Schaefer 1998; Sargent et al. 1999; Mañanós et al. 2008).

The FA composition of *C. ramada* muscles was distinct between estuary and freshwater contingents, with the FA trophic marker analysis supporting this result. These differences reflect the food and energy sources exploited in each environment (Almeida 1993; 2003) and reinforce the dietary influence on muscle FA profile. Specifically, the freshwater contingent showed a critical marker of freshwater epiphytic microalgae-like diatoms (Carassou et al. 2017), with a clear predominance of the n-7 FA family, which was attributed to the high levels of the relative amount of total n-7 MUFA, mainly palmitoleic acid. The C16:1n-7 is also the primary product of the Δ9 desaturase during the MUFA pathway and may serve as a vital energy source during larval development (Ortega and Mourente 2010). On what concerns the estuarine contingent, *C. ramada* displayed a higher relative amount of the n-9 FA family, which was associated with the substantial amount of oleic acid, a crucial FA in the β-oxidation process and an important source of potential metabolic energy (Tocher 2003). Our results are corroborated by another study in which the analysis of the FA composition of a distinct mugilid species, the freshwater mullet *Myxus capensis* (Günther, 1876), that also moves to freshwater, identified the C16:1n-7 and C18:1n-9 as the two major MUFA in muscle, representing 47% and 33%, respectively (Carassou et al. 2017). Ramos-Júdez et al. (2023) also reported similar results regarding the muscle FA profile of *Mugil cephalus* (Linnaeus, 1758) during ovarian development. Additionally, the estuarine contingent showed a higher relative amount of n-3 PUFA, such as C18 PUFA and mainly C20 PUFA, which constitute the principal PUFA in macroalgae. These results suggest a more significant influence of seaweeds and a more varied diet in terms of macroalgae and phytoplankton (Carassou et al. 2017), most notably dinoflagellates because either the ratio EPA/DHA < 1 or the higher relative amount of C18:4n-3, the major component of the lipids of most species of dinoflagellates (Napolitano 1999; Dalsgaard et al. 2003). The presence of several microbial FAs, albeit in smaller relative amounts, suggests that the availability of these FAs in the diet of both groups varies significantly (Bec et al. 2003; Dalsgaard et al. 2003; Jaschinski et al. 2008). Despite odd and branched-chain FAs being characteristic of bacteria, they can be found frequently in microbial mats composed of diatoms alone or diatoms in combination with bacteria (Lechevalier 1982; Kharlamenko et al. 1995). This highlights that seasonal changes in habitat and feeding behavior result in significant dietary shifts strongly influenced by the dominant food resources available in each area, leading to notable changes in fish condition and the muscle FA composition (Carassou et al. 2017).

At the end of the trophic migration (i.e., late summer), it is worth noting that the muscle of both contingents showed a reduction in unsaturated fatty acids, particularly HUFA when compared to the FA profile obtained at the beginning of the trophic migration (early spring). In turn, the gonads from that season revealed a high expression of unsaturated FAs, especially of the n-3 FA family, the most relevant HUFA. The LC-PUFA comprises EFAs (such as EPA and DHA) with a vital role in vitellogenin (Almansa et al. 2001; Dhurmeea et al. 2018; El-Moneem et al. 2022), primarily in the initial phases of oocyte development and maturation (Silversand and Haux 1995; Sorbera et al. 2001; Khajeh et al. 2017; Tong et al. 2017), and a source of energy during embryogenesis and early larval development. Therefore, our results are consistent and pointed out that unsaturated FAs have been mobilized from muscle and allocated to the gonads. Nevertheless, the gonads from freshwater had a clear predominance of n-3 FA metabolic pathway, including some of the FA precursors of DHA. Thus, despite that the freshwater contingent revealed a higher gonad dry weight and total lipid content, the EFAs present in the lipid pool did not show the high DHA retention we might expect from the more advanced macroscopic maturity stages of the gonads (predominantly stage IV). Conversely, mature *C. ramada* in stage III from estuaries showed lower GSI and lower total lipid content but revealed a higher DHA relative amount in their lipid profile. The FA profile observed in the gonads of the freshwater contingent could be associated with the fact that C. *ramada* follows the typical reproductive cycle of late autumn/early winter-spawning species. This cycle is characterized by a long stage III of maturity that lasts from the late winter to late summer, with high lipid accumulation in summer, and a very short stage V (Lajus and Alekseev 2019) that, in this specific case, is only achieved at sea (Almeida 1996). Hence, a natural delay of ovary development, similar to a phenomenon of dormancy, enables the adults to exploit and take advantage of the warm and food-rich summer/autumn period and adjust spawning and juvenile appearance during the most favorable conditions (i.e., Mañanós et al. 2008; Zarski et al. 2013; Lajus and Alekseev 2019). From the point of view of either migratory behavior or migratory physiology, early gonad maturation would be a process with high energy costs that could reduce the growth period and/or jeopardize reproductive success. Indeed, the very low relative amount of DHA and a gonad EPA/DHA ratio > 1 found in this study indicate that this condition might be related to the n-3 FA pathway and the processes of FA conversion by elongation and desaturation. Specifically, in the freshwater contingent, ALA makes up 24.49% of the total n-3 FA in the gonads, DPA contributes 16.50% of the total n-3 FA, and EPA constitutes nearly 32% of the total n-3 FA in the gonads. The low level of DHA in the freshwater contingent must be associated with the extent to which they can biosynthesize DHA from C18 or C20 PUFA precursors. It is well understood that the repertoire and function of genes encoding Fads and Elovl vary among fish species, thus determining the extent to which each species can biosynthesis C20–24 PUFA such as EPA, ARA, and DHA from C18 PUFA (Bell and Tocher 2009; Monroig et al. 2013 ).

From a species reproduction perspective, some studies state that the later stages of gonad development may require supplementary energy derived from feeding at the estuary (Koussoroplis et al. 2010; Mousa et al. 2018). Our findings emphasize that for *C. ramada*’s freshwater contingent, a pre-spawning period in the estuary is essential. This pre-spawning period not only guarantees a coordinated spawn and reduces predation rates but, most importantly, will be crucial to ensure that the individuals attain the appropriate ratios of EFAs. However, it is possible that some individuals may not reach the later stages of gonad development. As a result, we hypothesize that a fraction of these individuals may not participate in the species’ annual breeding cycle and remain in the estuary after the trophic migration. This is supported by ongoing multi-elemental otolith microchemistry analysis indicating that some individuals may lack annual spawning migrations (Unpublished data). These individuals could potentially serve as a stronghold for the population, suggesting a complex catadromous life cycle similar to what is observed in some anadromous species (e.g., Atlantic salmon (*Salmo salar* L.), Thorstad et al. 2010).

In contrast to the freshwater contingent, the population fraction with greater estuarine residency appeared to be in metabolic synchrony with the observed gonad maturation stages and the species’ reproductive cycle (González-Castro and Minos 2016; Ramos-Júdez et al. 2023). The existence of both continents enables *C. ramada* to take advantage of temporal windows of favorable conditions for both adult and offspring survival (Mañanós et al. 2008). This may also explain the extended spawning periods observed across *C. ramada* distribution (Bruslé, 1981), as well as the existence of protracted recruitment events (Bartulovic et al. 2007; González-Castro and Minos 2016), and the maintenance of a unique species metapopulation (Pereira et al. 2023). In this sense, besides the lower predation from piscivorous fish and having a low dietary overlap with other mugillids (Salvarina et al. 2018), migratory behavior to freshwater appears to promote a conditional reproductive strategy. Under unpredictable environmental conditions and stochastic events, it maximizes adult fitness while promoting a balance between ecosystem productivity and recruitment. Moreover, the maintenance of alternative life strategies within a population enables the reduction of the population’s exposure to drastic habitat changes at a particular location/time, and it also allows the exploitation of recently restored habitats (Anderson and Quinn 2007). Therefore, migration into freshwater is undoubtedly a vital component of a species’ life cycle with a notable impact on fitness and population maintenance.

This work provides valuable contributions to clarify the importance of freshwater habitats for a significant fraction of the population and its implication for the species’ fitness. From the point of species conservation and management, it highlights the species’ vulnerability to human-induced habitat alterations, such as dams and other obstacles to migration and to modification of physicochemical parameters related to global change (e.g., increasing temperature, pH, and oxygen saturation) that are known to modify the sources of FAs available (Hixson and Arts 2016).

Additional studies should be conducted to compare the reproductive status with the fatty acid profile, both polar and neutral lipids, to investigate FA allocation. On the other hand, there is a need to further investigate the obligatory character of spawning migration and how individuals’ migratory behavior and breeding patterns may change with ontogeny and environmental conditions. Hence, future studies focusing on otolith microchemistry may provide helpful insights to unveil the inter-annual variability and species’ behavioral plasticity.

### Supplementary Information

Below is the link to the electronic supplementary material.

Supplementary file1 (DOCX 15 KB)

## Data Availability

The datasets generated and/or analyzed during the current study are available from the corresponding author upon reasonable request.
