# Supplementary material for: Do catadromous thinlip grey mullet benefit from shifting to freshwater? A perspective from fatty acid signature analysis
Source: Fish Physiol Biochem. 2024 Feb 27;50(3):1093–108. doi: 10.1007/s10695-024-01322-9 (PMC11213746; doi:10.1007/s10695-024-01322-9)
Supplement: Supplementary file 1 — Supplementary file1 (DOCX 15 KB) [file 10695_2024_1322_MOESM1_ESM.docx]

# Supplementary material

| Supplementary material 1: Fatty acids considered for each trophic marker | | | | | | | | |  |  |
| --- | --- | --- | --- | --- | --- | --- | --- | --- | --- | --- |
| **Microbial** | | **Macroalgae** | | **Diatoms** | | **Dinoflagellate** | | | |  |
|  | C14:0i; |  | C18:2n-6; |  | C14:0; | |  | C18:1n-9; | | |
|  | C15:0i; |  | C18:3n-3; |  | C16:1n-7; | |  | C18:4n--3; | | |
|  | C15:0a; |  | C18:4n-3; |  | C16:2n-6; | |  | C22:6n-3; | | |
|  | C15:0; |  | C20:4n-6; |  | C16:3n-4; | |  | C22:5n-3; | | |
|  | C16:0i; |  | C20:4n-3; |  | C20:5n-3; | |  | C20:5n-3; | | |
|  | C17:0a; |  | C20:5n-3; |  | C16FA/C18FA; | |  | C16FA/C18FA; | | |
|  | C17:0i; |  | C18 PUFA; |  | C16 PUFA | |  | C16 PUFA; | | |
|  | C19:0; |  | C20 PUFA; |  |  | |  | DHA/EPA | | |
|  | Σi; |  | C16PUFA/C18PUFA; |  |  | |  |  | | |
|  | ΣIsso+anteiso |  | C16 PUFA; |  |  | |  |  | | |
|  |  |  | ΣC18PUFA + C20 PUFA |  |  | |  |  | | |
